# Characteristics of Head and Neck Injuries Among Pediatric Ice Skaters in the United States

**DOI:** 10.7759/cureus.93021

**Published:** 2025-09-23

**Authors:** Nick Sahlollbey, Huzaifa Saeed, Erin M Gawel, David Riccio, Maya Raghavan, Michele M Carr

**Affiliations:** 1 Otolaryngology-Head and Neck Surgery, University of Calgary, Calgary, CAN; 2 Anaesthesiology, College of Medicine, University of Saskatchewan, Saskatoon, CAN; 3 Otolaryngology, University at Buffalo Jacobs School of Medicine and Biomedical Sciences, Buffalo, USA

**Keywords:** child, facial lacerations, head injuries, ice skating, injury prevention, neck injuries

## Abstract

Purpose

Injuries sustained while ice skating are common; however, the prevalence, distribution, and anatomical location of head and neck injuries (HNI) are not well known. The purpose of this study was to describe patterns in ice-skating-related head and neck injuries in children.

Study design

This was a retrospective review.

Methods

We conducted a retrospective review of data involving children aged 18 years and younger from the National Electronic Injury Surveillance System, a public database containing information from approximately 100 emergency departments across the United States. We utilized data from January 2002 to December 2021. Data gathered included patient demographics, injury type, injury location, injury outcome, and year of incidence.

Results

A total of 2686 ice-skating injuries were identified. 1594 (59%) of those involved the face, neck, ear, chin, nose, forehead, or mandible. Of these, 1023/1594 (64%) occurred in males and 571/1594 (36%) in females; 1653 (62%) patients sustained a laceration. The most commonly affected site of HNI was the chin, with 911/1594 (57%) injuries. Injuries to the neck were most commonly a sprain/strain with 32 injuries, while those affecting the mandible were most commonly a fracture with 4 injuries.

Conclusion

Young ice-skaters are most likely to sustain lacerations or other injuries to the chin.

## Introduction

Ice-skating is a popular winter recreational sport. These activities place individuals at risk for acute and chronic injuries; nevertheless, there is relatively little data on injuries sustained while ice-skating [1].

The National Electronic Injury Surveillance System (NEISS) is a government-sponsored initiative that gathers information on injuries treated in hospital emergency departments (EDs) in the US [2]. The NEISS database contains data on the activity that caused the injury, the body part harmed, the specific diagnosis and basic treatment, and the injured person's demographic data.

The literature has shown that head and neck injuries (HNI) comprise a sizable fraction of all ice-skating injuries [3,4]. Nevertheless, it is unclear how these injuries are classified (e.g., contusions, lacerations, and hematomas) or which anatomical regions are involved. We hypothesized that the chin and mandible would be the most likely sites of ice-skating-related injuries in children. Therefore, we analyzed NEISS data on ice-skating injuries and described their characteristics, including diagnosis, anatomic location, and patient demographics. A comprehensive understanding will help direct efforts to prevent potentially disfiguring and debilitating injuries related to ice-skating.

## Materials and methods

Source

We conducted a retrospective review of data obtained from the NEISS in April 2023. The NEISS is a public database maintained by the United States Consumer Product Safety Commission and contains information from approximately 100 EDs. We acquired data on over 2,600 ice-skating injuries between January 2002 and December 2021. Because this research involved public deidentified data, the study was exempt from institutional review based on institutional policies.

Selection criteria

Our study population was isolated to contain ice-skating-related injuries. “Ice-skating-related injury” was defined as any accident while the individual had skates on. Non-ice-skating entries were excluded. We extracted 2712 records, and 26 were excluded. Narratives describing details of the injury were reviewed to ensure accuracy of age, diagnosis, and body part. To characterize facial injuries more specifically, extra codes were added for chin, nose, forehead, and mandible. These modifications were placed in a spreadsheet and utilized for subsequent analysis. Information about age, weight, sex, and race was examined.

Injury diagnosis (concussion, contusion/abrasion, fracture, hematoma, laceration, dental injury, internal organ injury, puncture, sprain/strain, hemorrhage, avulsion) and injury location (head, face, eyeball, mouth, neck, ear, chin, nose, forehead, mandible) were also analyzed.

NEISS was used to acquire data on over 2600 ice-skating injuries between 2002-2021. Data were reviewed by three members of the research team based on inclusion/exclusion criteria (see below). An ice-skating injury was defined as any accident that occurred while the individual had their skates on. Any entries that did not conform to this definition were excluded. Head and neck-related injuries included those affecting the face, neck, ear, chin, nose, forehead, or mandible, regardless of whether it was listed as the primary or secondary injury site. ‘Head’ injuries (Code 75) were not included - unless there was a second concomitant head and neck-related injury, such as a laceration - as this classification primarily corresponds to neurological injuries, such as cerebral contusion, subdural hematoma, and skull fracture.

We recorded age, diagnosis, and body part, corroborating the entries with the corresponding narrative. This step was performed to ensure that the recorded data accurately reflected the nature of the ice-skating injuries. Demographic variables occasionally included values coded as ‘unknown’ within NEISS (e.g., race), which we retained as their own category. No additional missing data were identified, and no imputation was required. Two members of the research team independently reviewed all narratives and coding decisions to ensure consistent application of inclusion and exclusion criteria, with discrepancies resolved by consensus. We consolidated data from various years into a single spreadsheet. This final dataset served as the foundation for executing descriptive statistics, enabling us to present a more holistic view of the trends and patterns in ice-skating injuries.

Statistical analysis

Data was merged, and descriptive statistics were performed. Chi-squared and Kruskal-Wallis tests were conducted to compare categorical and continuous variables. Statistical significance was set for a p-value less than 0.05. Data analyses were performed using Stata 15 software (StataCorp, College Station, USA) [5].

## Results

**Figure 1:**
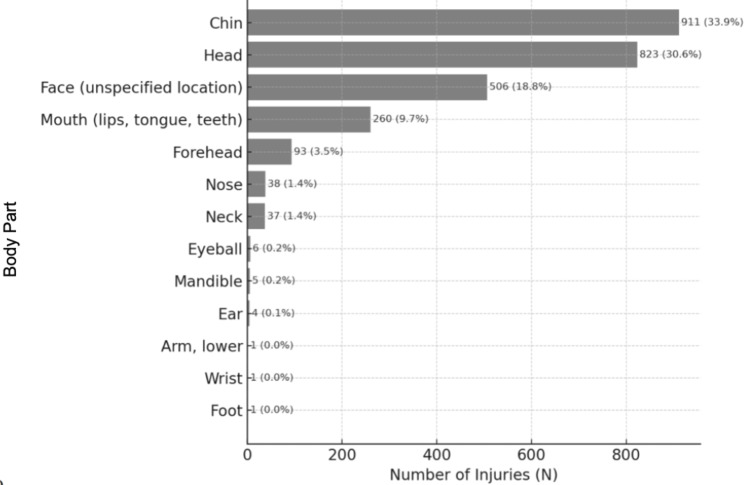
Anatomical Location of Injury 1594 (59%) of all injuries were head and neck-related (face, ear, neck, chin, nose, forehead, and mandible). N.B.: ‘Head’ injuries were not included in head and neck-related injuries, as this classification primarily corresponds to neurological injuries, such as cerebral contusion, subdural hematoma, and skull fracture.

The average age was 10 years (range 2 to 18 years), and two-thirds were male (Table 1). We included 2686 ice-skating injuries. Of these, 1594 (59%) involved the face, neck, ear, chin, nose, forehead, or mandible (Figure 1). Patients were most likely to sustain lacerations, with 1653 cases representing 62% of all injuries. Specifically, lacerations were the predominant injury to the face (N=421 out of 506 total facial injuries; 83%), ear (N=4 out of 4 ear injuries, 100%), chin (N=902 out of 911 chin injuries; 99%), and forehead (N=77 out of 93 forehead injuries; 83%). The chin (N=911/2686; 34%) and head (N=823/2686; 31%) were the most commonly injured body parts. Neck injuries were primarily sprains/strains, representing 32 out of 37 total neck injuries, or 87%, while mandible injuries were mostly fractures (N=4 out of 5 mandibular injuries; 80%). Nose injuries varied, with contusions or abrasions being the most common (N=17 out of 38, 45%) (Table 2). The majority of cases captured were in patients with a primary diagnosis of HNI (Table 3).

**Table 1:** Demographics

Demographic	Value
Age (years), mean ± SD	10.2 ± 3.8
Age (years), median [95% CI]	10 [9–10]
Weight (kg), mean ± SD	27.8 ± 27.8
Weight (kg), median [95% CI]	15.5 [15.5–15.7]
Sex	
Male, N (%)	1659 (62)
Female, N (%)	1027 (38)
Race	
White, N (%)	1342 (50)
Black/African American, N (%)	206 (8)
Asian, N (%)	68 (2)
Other, N (%)	100 (4)
Unknown, N (%)	970 (36)

**Table 2:** Head and Neck vs. Non-Head and Neck Injuries Chi-square test comparing distribution of head and neck vs. non–head and neck injuries across diagnoses: χ²(11) = 1548.3, p < .001. Head and neck injuries were more likely to be lacerations than non–head and neck injuries; neck injuries were most often sprains; mandible injuries were most often fractures. HNI: head and neck injuries

Diagnosis (Code)	All N (%)	All HNI (% of total)	Non-HNI (% of total)	Face (% of subsite)	Neck (% of subsite)	Ear (% of subsite)	Chin (% of subsite)	Nose (% of subsite)	Forehead (% of subsite)	Mandible (% of subsite)
Concussion (52)	20 (0.7)	1 (5)	19 (95)	0	0	0	1 (0.1)	0	0	0
Contusions, abrasions (53)	212 (8)	101 (48)	111 (52)	68 (13)	2 (5)	0	4 (0.4)	17 (45)	10 (11)	0
Fracture (57)	34 (1.3)	23 (67)	11 (32)	11 (2)	2 (5)	0	1 (0.1)	6 (16)	0	4 (80)
Hematoma (58)	29 (1.1)	14 (48)	15 (52)	4 (0.8)		0	0	3 (8)	6 (6)	0
Laceration (59)	1653 (61.5)	1416 (86)	237 (14)	421 (83)	1 (3)	4 (100)	902 (99)	10 (26)	77 (83)	1 (20)
Dental injury (60)	88 (3.3)	2 (2)	86 (99)	0	0	0	2 (0.2)	0	0	0
Internal organ injury (62)	610 (22.7)	1 (0.00 1)	609 (99.9)	0	0	0	1 (0.1)	0	0	0
Puncture (63)	1 (0.01)	1 (100)	-	1 (0.2)	0	0	0	0	0	0
Strain/sprain (64)	32 (1.2)	32 (100)	-	0	32 (87)	0	0	0	0	0
Hemorrhage (66)	3 (0.01)	3 (100)	-	1 (0.2)	0	0	0	2 (5)	0	0
Avulsion (72)	3 (0.11)	-	3 (100)	0	0	0	0	0	0	0
Other/Not stated (74)	1 (.001)	-	-	0	0	0	0	0	0	0

**Table 3:** Frequency and Percentage of Primary Versus Secondary HNI Diagnoses for Ice Skating-Related Injuries Frequency and percentage of primary and secondary diagnoses for injuries related to ice skating. The majority of cases captured were in patients with a primary diagnosis of HNI. HNI: head and neck injuries

Diagnosis Code	Diagnosis Description	Primary Diagnosis (%)	Secondary Diagnosis (%)	Total (%)
52	Concussions	20 (0.7)	15 (0.5)	35 (1.2)
53	Contusions, Abrasions	212 (7.5)	29 (1.0)	241 (8.6)
57	Fracture	34 (1.2)	2 (0.1)	36 (1.3)
58	Hematoma	29 (1.0)	3 (0.1)	32 (1.1)
59	Laceration	1653 (58.8)	27 (1.0)	1680 (59.8)
60	Dental Injury	88 (3.1)	8 (0.3)	96 (3.4)
62	Internal Organ Injury	610 (21.7)	22 (0.8)	632 (22.5)
63	Puncture	1 (0.0)	0 (0.0)	1 (0.0)
64	Strain or Sprain	32 (1.1)	13 (0.5)	45 (1.6)
66	Hemorrhage	3 (0.1)	1 (0.0)	4 (0.1)
71	Other/Not Stated	0 (0.0)	4 (0.1)	4 (0.1)
72	Avulsion	3 (0.1)	0 (0.0)	3 (0.1)
74	Dermatitis, Conjunctivitis	1 (0.0)	0 (0.0)	1 (0.0)

Of all injuries, patients were most likely to sustain a laceration (N=1653; 62%). Lacerations were the most common injury of the face (N=421/506; 83%), ear (N=4/4; 100%), chin (N=902/911; 99%), and forehead (N=77/93; 83%) (Table 2). The most commonly affected body parts overall were the chin (40%), followed by the head (31%) (Figure 1). Injuries to the neck were most likely to be a sprain/strain (87%). Mandible injuries were likely to be fractures (80%). Diagnosis of nose injuries varied but tended to be contusions or abrasions (45%) (Table 2).

**Figure 2:**
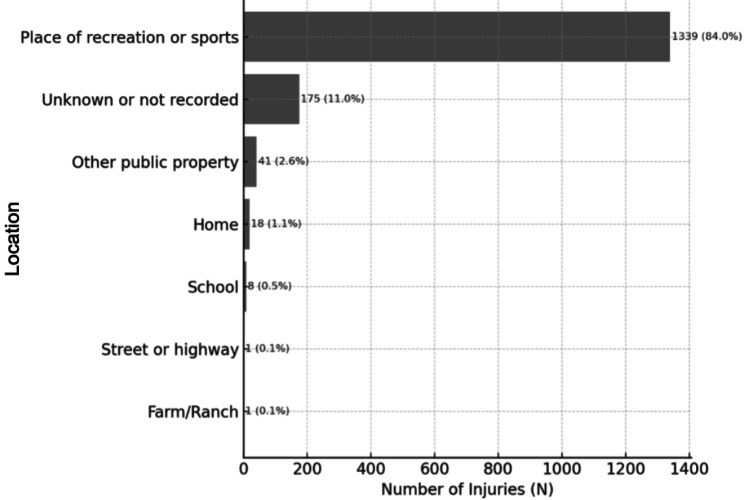
Geographical Location of Injuries χ²(6) = 2.65, p = .900. Body part injured was not associated with location. Values shown as N (%).

**Figure 3:**
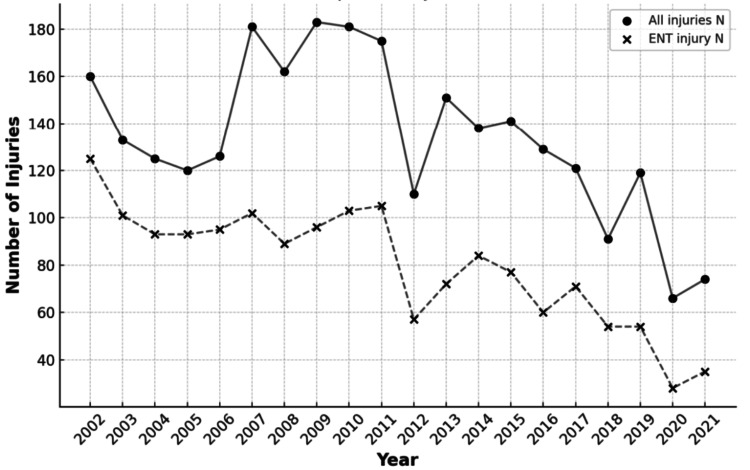
Trend of Injuries from 2002 to 2021 This figure illustrates a decrease in the total number of injuries (solid) and head and neck injuries (dashed) observed over the period. Linear regression analysis revealed a significant decrease in injuries reported over time (p<.001). ENT: Ear, Nose, Throat

Most head and neck injuries (84%) occurred at a place of recreation or sports (Figure 2). Although a proportion of narratives failed to report the geographical location where the injury was sustained, the body part injured was not associated with the place the injury occurred (p = .900). Individuals with head and neck injuries were likely to be treated and released or examined and released without treatment (99%) and unlikely to leave without being seen (0.6%) or be admitted for hospitalization (0.3%) (Table 4). Children sustained more head and neck injuries than non-head and neck injuries (p = .001). Most injuries were sustained by males (62%), who were more likely to sustain head and neck injuries (64%) than females (35%) (odds ratio = 1.284) (Table 5). Linear regression analysis revealed that fewer head and neck injuries were reported over time (p< .001, Figure 3). There were no reported deaths.

**Table 4:** Outcomes of Ice-Skating Injuries Chi-square test comparing disposition of head and neck vs. non–head and neck injuries: χ²(4) = 38.4, p < .001. Reject the null hypothesis that disposition is independent of body part (head and neck vs. non-head and neck). Head and neck injuries are more likely to be treated and released, not leave without being seen or admitted for hospitalization.

Disposition	All N (%)	Head and neck, N (%)	Comment
Treated and released, or examined and released without treatment (also includes one transfer for treatment to another department of the same facility without admission)	2620 (98)	1578 (99)	-
Treated and transferred to another hospital	7 (0.3)	3 (0.2)	3-Chin
Treated and admitted for hospitalization (within same facility)	16 (0.6)	4 (0.3)	3-Face, 1-Forehead
Held for observation (includes admitted for observation)	11 (0.4)	0 (0)	-
Left without being seen, left against medical advice, left without treatment, or eloped	32 (1.2)	9 (0.6)	2-Face, 5-Chin, 2-Forehead

**Table 5:** Relationship Between Demographics of Patients and Anatomical Location of Injures Kruskal–Wallis tests were used for continuous variables (age, weight). A chi-square test compared sex distribution (male vs female). Therefore, the test statistic and p-value are shown in the male row only. Head and neck injuries occurred more often in younger children. The sex distribution differed significantly, with a higher proportion of males and a lower proportion of females sustaining head and neck injuries compared with non–head and neck injuries.

Demographic	Head and neck body part	Non–head and neck body part	Test statistic	p–value
Age (years), median [95% CI]	9 [9–10]	10 [10–11]	H = 10.9	.001
Weight (kg), median [95% CI]	15.5 [15.5–15.8]	15.4 [15.4–15.7]	H = 2.9	.09
Sex – Male, N (%)	1023 (64)	636 (58)	χ²(1) = 9.4	.002
Sex – Female, N (%)	571 (35)	456 (42)	–	–

## Discussion

Our analysis of NEISS found that injured pediatric ice skaters frequently sustain injuries to the face, particularly lacerations or other injuries to the chin/mandible. These findings add to the knowledge base regarding head and neck injuries sustained by children while ice skating [1,3,4,6].

The relatively large number of injuries documented in this study highlights the substantial public health risk that ice-skating injuries represent, particularly among children. The high rate of laceration may be attributed to the mechanism of ice-skating falls, where the fast impact against the hard ice surface can instantly cause skin and soft tissue injuries. Utilizing helmets with face shields (i.e., clear visor) or guards (i.e., wire/metal grid) may lower this risk, highlighting the necessity for targeted injury prevention strategies [4]. The high frequency of head and chin injuries, which make up 34% and 31% of all injuries, respectively, calls for further attention. The adoption of safety precautions, such as the use of protective gear like helmets, face shields or guards, and mouthguards, as well as ensuring that such gear is fitted adequately and worn properly, may help to reduce the frequency of these injuries.

Neck injuries were more likely to result in sprains or strains compared to mandibular injuries, which aligns with Dhodapkar et al’s findings that head, face, and neck injuries are predominant in ice-skating [6]. These distinct injury patterns may be related to the biomechanics of ice-skating injuries, where the neck may be subjected to fast twisting motions and the jaw may quickly strike the ice. Previous studies further emphasize the higher incidence of head injuries in ice skaters compared to other skating activities, underscoring the need for targeted protective measures [3,4]. The implementation of suitable safety gear and teaching of correct falling techniques could potentially prevent these injuries [8,9].

Our findings confirm the results of previous studies showing that boys sustain more injuries than girls in skating-related activities [10-12]. This gap could be explained by several factors, including potential disparities in skating skill, aggression, risk-taking behavior, and use of protective equipment. The higher risk of head injuries in ice skating also suggests the necessity for enhanced safety protocols and equipment, particularly helmets, to mitigate these risks [4]. Our study not only corroborates the existing literature on the prevalence of head and neck injuries in ice skating but also highlights the critical need for preventative strategies tailored to the unique dynamics of this sport.

We found that 84% of HNI occurred in sporting or recreational venues, highlighting the need for improved security and control at these locations. Additionally, even though there were many injuries, most patients received care and were later released, consistent with the fact that most of these injuries were not life-threatening. Using linear regression analysis, we found that the rates of HNI decreased over time (p<.001), which may be a result of improved safety measures or adjustments to leisure activities (Figure 1). It is nevertheless critical to devote attention to trends and implement prevention measures to ensure that this decline in injuries continues.

We observed a significantly higher number of primary diagnoses compared to secondary diagnoses for head and neck injuries related to ice skating. This discrepancy can be attributed to several factors inherent to the NEISS database and emergency department reporting practices. Primarily, emergency department staff focus on documenting the most severe and immediate injuries requiring treatment, often resulting in the prominent recording of primary diagnoses. Secondary injuries, unless critically impacting the treatment plan, may not be consistently documented. Additionally, the NEISS database’s structure prioritizes the capture of primary diagnoses for statistical purposes, which may lead to the underreporting of secondary injuries

This study has limitations to acknowledge. First, NEISS data is collected from ED visits. Therefore, patients who seek care outside of an ED or received no treatment were not included, under-representing the total number of ice-skating injuries. NEISS data used the product-code system, which restricts the accuracy with which we can predict population rates and trends. We did not segregate injuries sustained while playing ice hockey, a contact sport associated with injuries [13-15]; therefore, we cannot determine the risk of injuries sustained during recreational skating, although the database has a separate code for ice hockey. Narratives often lacked details on the injury mechanism, protective equipment usage, or the skating experience of the injured individuals; these factors were not considered in our analysis.

## Conclusions

In conclusion, this study utilizes a large national database to provide critical insights into the patterns and characteristics of head and neck injuries among pediatric ice-skaters. The prevalence of lacerations, particularly to the chin, underscores the need for enhanced safety measures, including the use of protective headgear. The findings highlight the public health implications of ice-skating injuries and advocate for the adoption of targeted prevention strategies to mitigate the risk of these injuries, especially in recreational and sporting venues.
